# A Hard Voting Policy-Driven Deep Learning Architectural Ensemble Strategy for Industrial Products Defect Recognition and Classification

**DOI:** 10.3390/s22207846

**Published:** 2022-10-16

**Authors:** Okeke Stephen, Samaneh Madanian, Minh Nguyen

**Affiliations:** Computer Science & Software Engineering, Auckland University of Technology, Auckland 1010, New Zealand

**Keywords:** deep learning ensemble, visual inspection, defect recognition and classification, voting policy, convolutional neural networks, industrial products

## Abstract

Manual or traditional industrial product inspection and defect-recognition models have some limitations, including process complexity, time-consuming, error-prone, and expensiveness. These issues negatively impact the quality control processes. Therefore, an efficient, rapid, and intelligent model is required to improve industrial products’ production fault recognition and classification for optimal visual inspections and quality control. However, intelligent models obtained with a tradeoff of high accuracy for high latency are tedious for real-time implementation and inferencing. This work proposes an ensemble deep-leaning architectural framework based on a deep learning model architectural voting policy to compute and learn the hierarchical and high-level features in industrial artefacts. The voting policy is formulated with respect to three crucial viable model characteristics: model optimality, efficiency, and performance accuracy. In the study, three publicly available industrial produce datasets were used for the proposed model’s various experiments and validation process, with remarkable results recorded, demonstrating a significant increase in fault recognition and classification performance in industrial products. In the study, three publicly available industrial produce datasets were used for the proposed model’s various experiments and validation process, with remarkable results recorded, demonstrating a significant increase in fault recognition and classification performance in industrial products.

## 1. Introduction

Industrial produce vision inspection [1,2] evaluates industrial part attributes to determine the conformity of production output to design specification and quality standards by providing insights into whether a manufactured product is accepted, reworked, or rejected. It involves the identification of defects or product deviations from an established design specification or standards. The inspection process assists industries in retaining a high reputation, reducing production material wastage and unnecessary production process interruptions, eliminating customer dissatisfaction, and sold goods return avoidance. Quality control is essential in delivering customers’ demands, and it deals with detecting problems and undertaking corrective action, in which the inspection process plays a critical role.

In the industrial setting, quality control is performed pre- and post-product manufacturing, as well as the manufacturing ecosystem completion to ensure the consistency of the outgoing product to quality standards and attainment of user expectations [3,4]. The visual inspection cycle is a sequential process that consists of the defect search process, recognition, and decision making. Furthermore, the manual industrial product defect inspection process is classified into four categories: (1) production monitoring, whereby an inspector typically monitors the production process to observe deviations from standards; (2) examination, where defects in products are searched for by the inspectors; (3) measurements, where the inspectors deploy tools and instruments to draw conclusions on the status of products; (4) patrolling, where inspectors change positions to check and organize works. However, many factors affect the manual inspection methods as aforementioned and thus necessitate the adoption of intelligent techniques.

**Figure 1:**
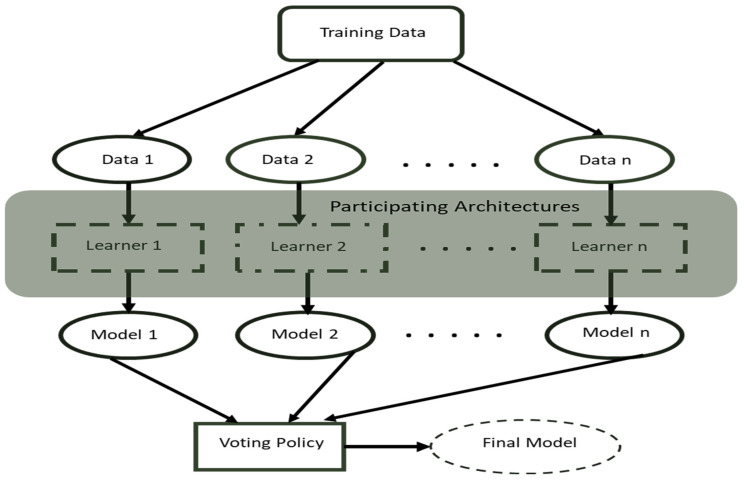
A cross-section of the ensemble model workflow with voting policy.

One such intelligent method is the artificial deep learning method that has recently drawn the significant interest of researchers. Deep learning [5] has been impactful in different research fields involving object detection [6], object segmentation [7], image classification [8,9], and speech recognition [10]. Deep learning provides more efficient and autonomous feature extraction than the manual feature extraction method and thus serves as a substitute in recent related scientific works. Also, deep ensemble learning, an advanced deep learning method, has contributed immensely to the enhancement of the deep learning-based high-level image frames feature computations and learning [11,12,13]. Therefore, in this work, an improved optimal deep learning method that deploys a voting policy strategy (see Figure 1), derived using three key main viable deep learning attributes such as architecture optimality, performance efficiency, and well accuracy, is introduced.

## 2. Related Works

In recent times, researchers have made tremendous efforts to develop intelligent vision models to recognize and classify defects in industrial products through the exploitation of different available industrial datasets for task-specific models. Wang, et al. [14] proposed deep learning and Hough transform-based intelligent vision models to inspect industrial products for defect recognition for prompt actions. The proposed model detects faulty products using three main activities: preprocessing input images, extracting regions of interest, and an image identification process. In another work, a textile quality control model presented by Jiang and Wong [15],Jeyaraj and Nadar [16] deployed computer vision models backed by a deep learning technique to scan and detect fabric defects, and Liong, et al. [17] used a deep learning architecture framework to classify and segment defects on leather surfaces. Furthermore, Liqun, et al. [18] introduced an enhanced VGG16 [19] to detect defects in vehicle parts for six major categories of vehicular components.

In continuation of deep learning frameworks on defect recognition, an automatic surface defects recognition model for steel strip production quality control using an effective compact convolutional neural network [9,20,21] to learn the low-level features present in the steel strips with the aid of multiple receptive fields was proposed in a study by [20,21]. In another related work, [22] proposed a lightweight Concurrent Convolutional Neural Network (ConCNN) to learn and classify steel surface multi-scale features for rapid real-time defect visual inspection and quality control. Also, a welding X-ray image frame defect classification was conducted using varied deep learning networks’ parameters and hyper-parameters in conjunction with other image processing algorithms such as Canny edge detection and adaptive Gaussian threshold methods [23]. In railway tracks, SqueezeNet [24] and MobileNetV2 [25] deep learning model architecture features were fused to obtain a lesser computationally expensive but faster model for defect spotting during railway tracks’ real-time visual inspection process.

Furthermore, a deep learning-based model for defect classification on imbalanced multi-label surface defects uses three strategies: transfer learning, imbalanced sampler, and fussy-fusionNet. These strategies were proposed to enhance and boost the classification performance of steel production quality control with reduced model complexity and better latency [26]. For the recognition of defects in multiple vital units of electrical components, Zhao, et al. [27] introduced a multi-stage pipeline for defect detection (MPDD) based on deep learning with region proposed network (RPN) anchor mechanism for image frame features fusion for the improvement of the proposed models’ defect detection performance accuracy and a conventional CNN for defect classification with a combination of super-resolution schemes for the defect classification performance improvement. In a photovoltaic (PV) cell production line, a deep learning framework was deployed to determine the presence or absence of defects using electroluminescence image frames in order to reduce the tedious work involved in the identification of faults in PV and to enhance the quality control process [28].

Chaudhary, et al. [29] in their work, proposed a method that combines five printed circuit boards (PCB) image frame processing stages such as: image registration [30], preprocessing [31], image segmentation [32], defect detection and classification to develop a robust rotation, scale and translation in-variant inspection model. In another related work, Kim, et al. [33] proposed a skip-connect convolutional autoencoder for the decoding of typical non-defect PCB images from defect images after training. Also, a you-only-look-once (YOLO) deep learning model was proposed to perform quality inspection of PCBs with an accuracy of about 98.79% [34]. Bhattacharya and Cloutier [35] proposed an end-to-end deep learning-based method to classify the PCB defects using Faster Region Convolutional Neural Network (FRCNN) in conjunction with the You-Only-Look-Once (YOLO), RetinaNet and ResNet50 as benchmark models. Using a similar model used by Kim, Ko, Choi and Kim [33], Khalilian, et al. [36] proposed a method that performs the detection of different types of defects on PCBs and equally localizes them with an accuracy of about 97.5%.

For recent works on detecting defects on pistons, an adaptive deep learning model was proposed by Tang, et al. [37]. Their proposed model used pressure signals from hydraulic piston pumps to perform defect diagnosis on the pistons. Wang, et al. [38] introduced a deep belief network, a variant of a deep learning model to detect and classify multiple defects from axial piston pumps to enhance the reliability and safety of the hydraulic system. Furthermore, an improved CNN model obtained from the modification of a proposed CNN’s cost function was introduced to diagnose defects on centrifugal pump components by deploying an analytical wavelet transform (AWT) algorithm to produce greyscale acoustic image frames through the acoustic signals processing and entropy-based divergence function for the minimization of redundancy during the CNN hidden layer activation process. Another related work introduced an intelligent-based model using a CNN model and wavelet analysis strategy to extract and classify faults on hydraulic piston pumps [39,40].

The casting production defect classification is not left out in the recent advancements in the use of deep learning methods to detect faults in industrial products. Lin, et al. [41] proposed a weakly-supervised CNN for casting X-ray image frame defects recognition. In another related study, Duan, et al. [42] deployed an enhanced You Only Look Once (YOLOv3)-based deep learning model that is robust and rapid in process with high detection efficiency to recognize casting defects and eliminate the issues associated with manual defect inspection methods. An automated method that used different CNN model architectures to perform localization of casting X-ray image defects through the transfer learning approach proposed by Ferguson, et al. [43]. Furthermore, Aluminum alloy casting defect detection based on CNN models was proposed in Mery [44], Nikolić, et al. [45]. In continuation, a deep, dense CNN framework proposed by Wu, et al. [46] utilized DenseNet121 as a backbone network to extract discriminative features from the casting images for accurate casting defect recognition.

Even though these are works on industrial product defect recognition and classification, an attempt is yet to be made to use the deep learning ensemble-based voting policy strategy, which we deploy in this work. The final objective of our research is to achieve optimal models for industrial defect identification and classifications.

## 3. Theoretical Background

### 3.1. Deep Learning Architectures

The deep learning model architectures selected for the competitive voting ensemble learning strategy for the industrial defect recognition and classification include a base convolutional neural network architecture [47], Inceptionv3 [48], Xception [49], DenseNet [50], MobileNet [51], and ResNet50 [52] architectures. The base CNN model consists of four main layers with feature extractors comprising of conv2 × 3, 32; conv2 × 3, 64 and conv2 × 3, 128 layer sizes, respectively, with a max-pooling layer of size 2 × 2 in-between the first and the second layers and Relu activator between the first three layers, as shown in Table 1. All the layers except the last layer use stride 2. The convolution and max-pooling operation outputs are assembled into feature maps of 45 × 45 × 32 and 22 × 22 × 32 sizes for the initial layer and 11 × 11 × 64, and 5 × 5 × 64 sizes for the second layer, respectively. The flatten layer with an output size of 1600 feature maps, the following dense layer with 128 output shape, and then the final dense having 1 output shape since the industrial product defect classification is a binary classification problem.

Furthermore, the Inceptionv3 [48] originated from the family of the Inception architecture with several enhancements such as the introduction of label smoothing strategy, 7 × 7 convolutions factorization, and the integration of an auxiliary classifier that passes label information to a lower ebb of the network. On the other hand, the Xception network [49] substitutes the Inception modules from the original Inception network architecture with depthwise separable convolution layers for efficient image feature processing and learning. In continuation, the DenseNet [50], exploits dense connections among layers in the architecture via dense blocks to improve the networks’ image feature learning capabilities, and individual layers in the network acquire extra inputs from all prior layers which are sent to the feature maps of all successive layers. For edge or compact device model deployments, a portable or lightweight deep neural network referred to as MobileNet [51] uses depthwise separable convolutions to process image features for a significant reduction of the network parameters. Finally, the last network architecture considered for the work is the ResNet50 [52], which is 50 layers deep. The networks’ residual blocks, together with bottleneck blocks, are stacked over one another to formulate the network.

### 3.2. Deep Learning Model Ensemble and Voting Policy Strategy

A typical deep learning ensemble method combines the outcome of multiple separate models to produce better performance results than an individual model. The objective of the proposed deep learning ensemble concept is to classify the industrial product input image frames efficiently and optimally into any *b* binary classes. The proposed method consists of a fixed number of deep learning architectures whereby each model obtained from the corresponding architecture takes an input and produces corresponding probabilities of a good or faulty product. The introduced method uses the voting policy to produce the best model depending on the aforementioned viable network characteristics.

The voting policy is based on the hard-voting ensemble approach, which is appropriate for model frameworks that produce distinct outputs. In other words, our hard voting method computes the predicted categories for each individual participating network. Unlike the conventional hard voting method, which votes on the probabilistic outcome of the input images each time the model is run, our introduced system votes on the performance of the trained models based on the three crucial aforementioned characteristics (model optimality, efficiency, and accuracy) and suggests the overall best model for subsequent deployment. The optimality and efficiency of the proposed model is reflected in the output weight of the voted model. The voted model’s weight is expected to be low, although it may not be the lowest, as we try to strike an equal balance between optimality, accuracy, and efficiency. Thus, this causes the voted model to rapidly and accurately classify defect and non-defect products, unlike other competing models with a high tradeoff between accuracy and latency and lag in production. This crucial factor makes the voted model more suitable for inferencing on low-cost devices.

The introduced ensembled strategy consists of several tunable parameters in the individual competing architectures and the voting policy. The tunable parameters control the learning process of the CNN models and determine the performance of the resulting usable model. Since we needed to maintain consistency and give an equal chance to the competing models, we left the tunable parameter values constant across the participating models. Two factors are considered to conduct the voting process, i.e., model efficiency and performance accuracy, as mentioned earlier. For one model to be regarded as best compared to the other, these two criteria must be attained to produce an improved overall model. This adopted voting concept partially adheres to the evolutionary algorithm model, and the method begins by producing a set of solutions, each adhering to the set ensemble criteria and configuration. All participating models were trained separately before the voting process was triggered for optimal computational resource utilization and to avoid overwhelming our computing resources while training the participating models together.

## 4. Materials and Methods

The experiments were run on a high-end computer with two GPU cores of one 12 GB video card each, 32 Gigabytes of RAM, Linux operating system, TensorFlow and Keras open-source deep learning libraries, and other relevant python modules such as Sk-learn, NumPy, etc.

### 4.1. Datasets

Three datasets were used to train, validate, and test the proposed model. The first dataset is the printed circuit board (PCB) industrial dataset [53], which originally consists of 1500 image pairs of the defect and non-defect images. The data were collected from a linear scan CCD within 48 pixels per 1-millimetre resolution. The defect and non-defect images were cross-checked manually and cleaned for use in the model training. The images were reduced to 1295 samples to strike an equal balance between the defect and the non-defect class and split into 892 samples for training, 223 samples for validation, and 180 samples for testing the proposed model.

Furthermore, the second dataset used in the experiments is the industrial mechanic component piston images that contain non-defect and defect (broken, shaped out, fallen, rust stains, greasy and oily) image classes [54]. The data were collected during the production of AC’s pistons and consists of 285 samples. In our experiments, the dataset was split into 173 training samples, 42 samples for the validation process and 70 samples for testing the proposed method. Finally, the third dataset used in this work is the industrial casting data obtained during the manufacturing of submersible pump impellers and publicly made available to researcher to build visual inspection models to detect defects and non-defect in them. The dataset originally contained 7348 image samples with 512 × 512 grayscale sizes [55]. To maximize our computing resources, the images were reduced to 300 × 300 grayscale sizes without affecting the quality of the images. A total of 4644 samples were assigned to the training set, 1989 were allocated to the validation set, and 715 reserved for the testing process. To ensure consistency across the overall experiment, all the datasets were normalized.

### 4.2. Experimental Procedure

To ensure fair competition among the selected models, equal parameters and hyperparameters were used across the pre-trained model architectures for fine-turning and retraining each of the models except the built conventional CNN model using the industrial production datasets. For instance, an ImageNet’s weight was used across the models while the top of all the models was sliced out. A 1 × 1 “ZeroPadding2D” padding, together with an extra Conv2D 1024 × 3 × 3 layer and a Relu activation function, were deployed across all the participating models. Furthermore, a “GlobalAveragePooling2D” and a 0.5 dropout rate were equally used across the models before the dense prediction layer with a sigmoid function across the models. The conventional models’ architecture is described in the previous section. During the actual training processes of all the participating models, an epoch of size 30 with a batch size of 32 was used in all the models, while the industrial PCB model inputs image size were set at 200 × 200, and the casting data input adjusted to 300 × 300, and the piston model inputs set at 90 × 90. In continuation, a learning rate scheduler of 1 × 10^−3^ × 0.9, binary cross entropy loss, and an Adam optimizer of 1 × 10^−4^ learning rate were utilized to train and validate all the models. After all the models were trained and validated separately and accordingly, the obtained results were subjected to the hard voting policy with respect to the three model viability criteria set for the study before the final best models for each of the datasets were generated.

## 5. Results

As mentioned in the previous section, 70 samples of the AC piston data, 715 submersible pump impeller casting data, and 180 samples of the PCB data were reserved for testing the proposed faulty and non-faulty industrial products recognition and classification model. The accuracy metric and the specificity, sensitivity, and Cohen kappa score estimation matrices of classification models were employed to evaluate the proposed model thoroughly. The specificity and sensitivity scores fit the statistical measurements dealing with binary classification tasks such as the various industrial datasets used in this study. In the context of this investigation, sensitivity deals with the capacity of the introduced model to correctly identify products with faults. On the other hand, the specificity score estimation metric is concerned with the capacity of the proposed model to correctly identify products without defects, while the Cohen kappa score computes the inter-rater reliability of the introduced model.

**Figure 2:**
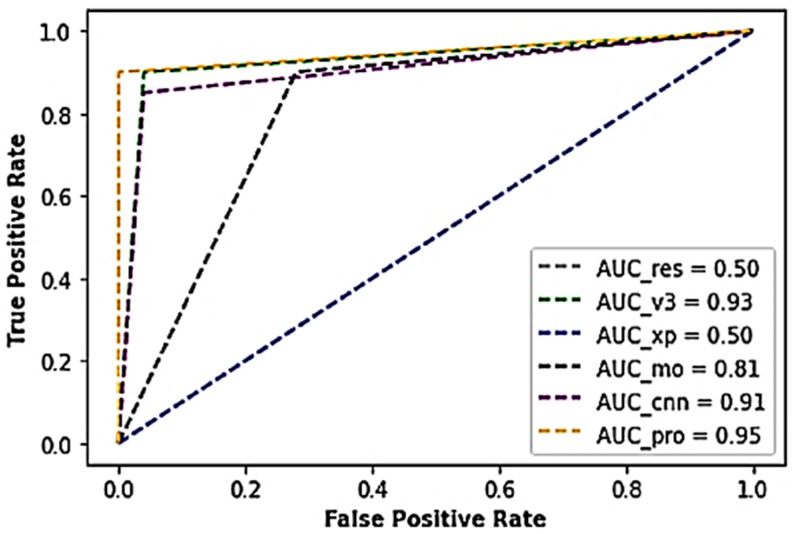
A cross-section of the AUC-ROC results of the industrial AC Piston dataset.

Table 2 illustrates the results of the various experiments conducted during the investigation using the industrial AC Piston dataset. As observed in the table, the voted model yielded an exceptional performance above 97% accuracy. Also, outstanding performance with approximately 90% sensitivity score, 100% specificity score, 93% Cohen kappa score, and a minimal test loss of about 5.24 × 10^−2^ was recorded compared to the conventional CNN model and Inceptionv3 models that outputted 9.29 × 10^−1^ and 9.43 × 10^−1^ accuracies, respectively. In the voting process, the Xception and MobileNet yielded lesser performances with accuracy scores above 70% with higher test loss scores of 5.04 × 10^−1^ and 1.84, respectively. Although not the lowest in terms of the outputted weight sizes, the voted model was considered for optimality and efficiency because there is a tradeoff between the accuracy and the weight output, as indexed in Table 2. In addition, the proposed model yielded an AUC of 0.95, while the ResNet model outputted the least AUC score of 0.50, as shown in Figure 2.

**Figure 3:**
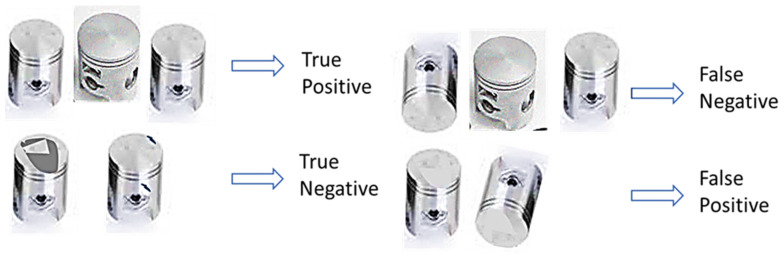
True positive, false negative, true negative, and false positive samples of the AC pistons.

**Figure 4:**
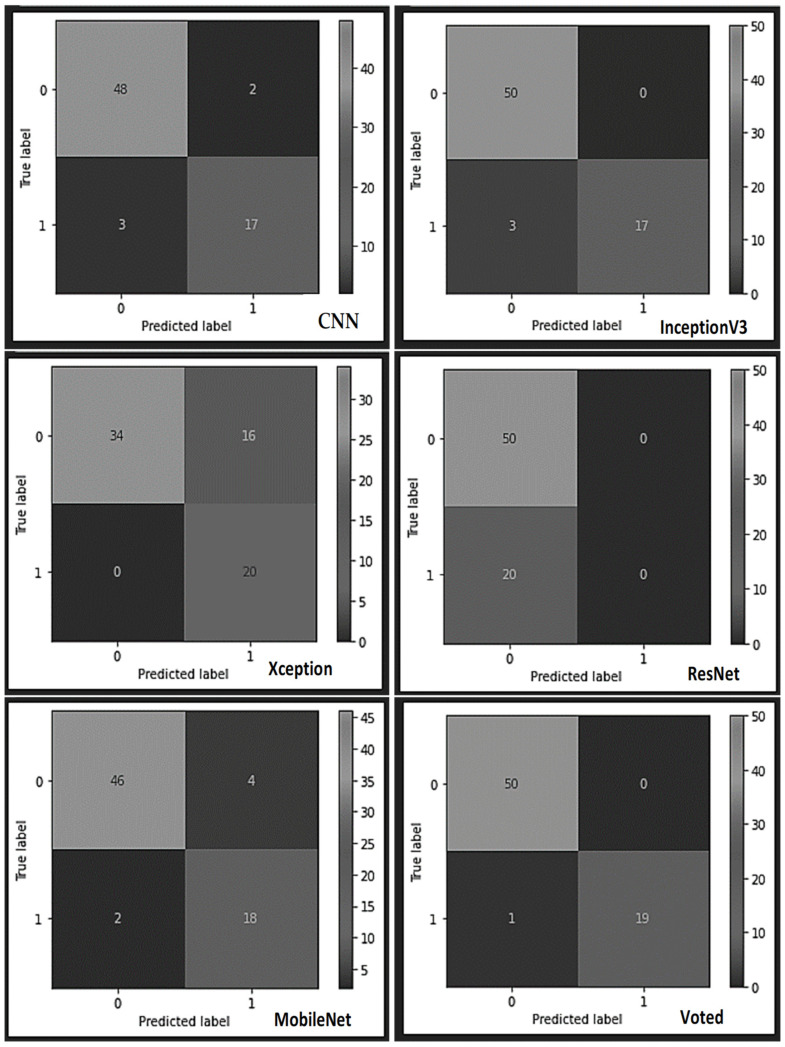
The various confusion matrices obtained from each learner using the industrial AC Piston dataset.

A more detailed analysis of the results obtained from the individual learners and the proposed model (see the confusion matrix table in Figure 3 and Figure 4) indicates that the constructed typical CNN model correctly classified 48 piston samples as non-defect pistons from the reserved test samples and misclassified two samples as defect pistons. Also, the CNN model appropriately classified 17 samples as faulty pistons from the bad piston samples and misclassified 3 samples as non-faulty samples. Conversely, the InceptionV3 model misclassified none of the 50 test samples of the good pistons but misclassified three samples of the faulty piston test samples as good out of the 20 faulty piston test samples. Furthermore, the Xception model misclassified 16 test samples of the good pistons as bad out of the 50 samples of the non-defect pistons while classifying all the 20 faulty test samples appropriately. According to the confusion matrix table, the ResNet learner displayed unreliable mixed results, while the MobileNet misclassified four samples out of the 50 good samples as bad, and appropriately classified 18 samples as bad samples. Finally, the voted model yielded superior performance by correctly classifying all 50 non-defect samples as good and misclassifying only one sample of the faulty piston test samples as good.

**Figure 5:**
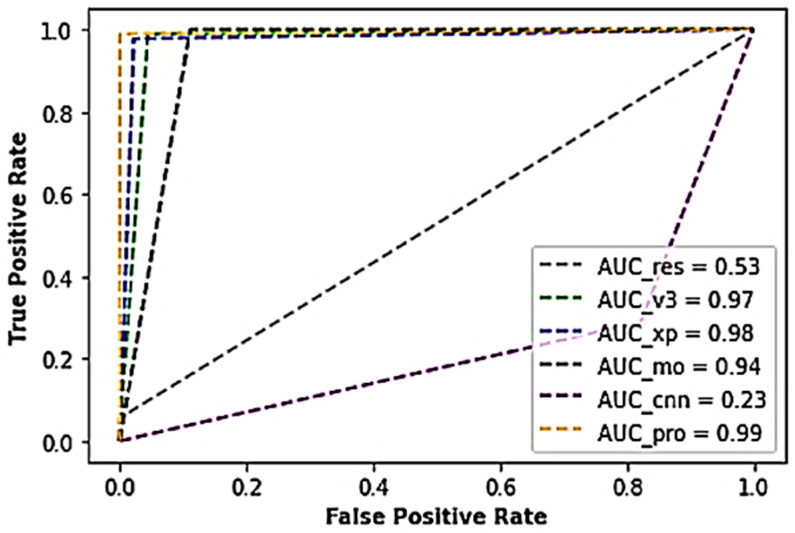
A cross-section of the AUC-ROC results of the industrial PCB dataset.

Furthermore, Table 3 represents the results obtained from the various experiments performed during the study using the industrial PCB dataset. As shown in the table, the voted model returned a superior performance of above 99.4% accuracy, about 98.9% sensitivity score, 100% specificity score, 98.9% Cohen kappa score, and a nominal test loss of about 3.80 × 10^−2^, beating the conventional CNN model and the other competing models. The traditional CNN model and the ResNet50 architecture yielded poor performances with accuracy scores of about 23.3% and 52.8%, with higher test loss scores of 1.43 and 7.15, respectively. Also, the voted model yielded an AUC score of 0.99, while the ResNet model outputted an AUC score of 0.53, Xception 0.98 AUC score, Inceptionv3 0.97 AUC score, typical CNN with 0.23 AUC score, and MobileNet with 0.94 AUC score, as shown in Figure 5.

**Figure 6:**
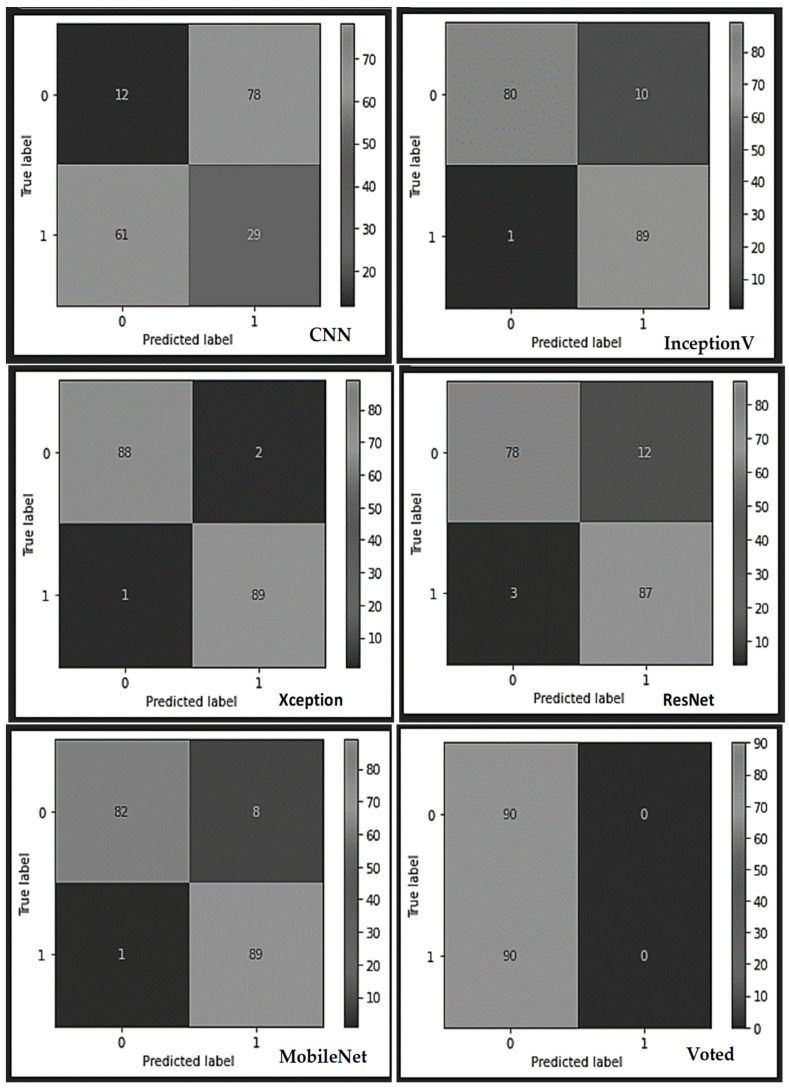
The various confusion matrices obtained from each learner using the industrial PCB dataset.

As indexed in Table 3, there exists a tradeoff between the accuracy and weight output of the voted model more than the other competing models. A further breakdown of the results obtained from the individual learners and the voted model using the PCB dataset (see the confusion matrix table in Figure 6) indicates that the built typical CNN model correctly classified 12 PCB test samples as non-defect and misclassified 78 samples as defect samples, thereby yielding overall abysmal performance. The CNN model also misclassified 29 samples as non-faulty and correctly classified 61 samples as faulty samples. Similarly, the Resnet model misclassified 12 samples as faulty class out of the 90 non-faulty test samples of the PCBs while misclassifying 87 samples as non-faulty instead of faulty samples. However, the InceptionV3 model correctly classified 80 test samples of good PCBs and misclassified 10 samples as bad ones. On the faulty samples of the PCBs, the InceptionV3 misclassified a whopping 89 samples while correctly classifying only one sample. Furthermore, the Xception model misclassified 89 test samples of the bad PCBs out of the 90 samples while correctly classifying 88 non-faulty test samples and misclassifying only two samples. In continuation, the MobileNet displayed a similar performance like the InceptionV3 model by misclassifying 89 samples out of the 90 bad samples and appropriately classifying 82 samples of the good samples. Finally, the voted model produced an exceptional performance by correctly classifying all the 90 non-defect and defect samples of the PCBs.

Finally, the outcome of the experiments run with the submersible pump impeller casting dataset during the study is presented in Table 4. As illustrated in the table, the voted model returned a better performance accuracy of about 99.90% with approximately 99.60% sensitivity score, 100% specificity, a Cohen kappa score of approximately 99.70%, and a minimal test loss of about 6.44 × 10^−3^, thereby beating the other competing models. All the models involved in the competition yielded sensitivity, specificity, Cohen_kappa, and accuracy scores above 99%, except the ResNet50 model, which returned a sensitivity of about 98.90%, 99.60% specificity, 98.50% Cohen_kappa, and an accuracy score of 99.30%, respectively. Also, the voted model produced the second lowest weight while maintaining high accuracy and thus was considered the most optimal and efficient by the voting policy.

**Figure 7:**
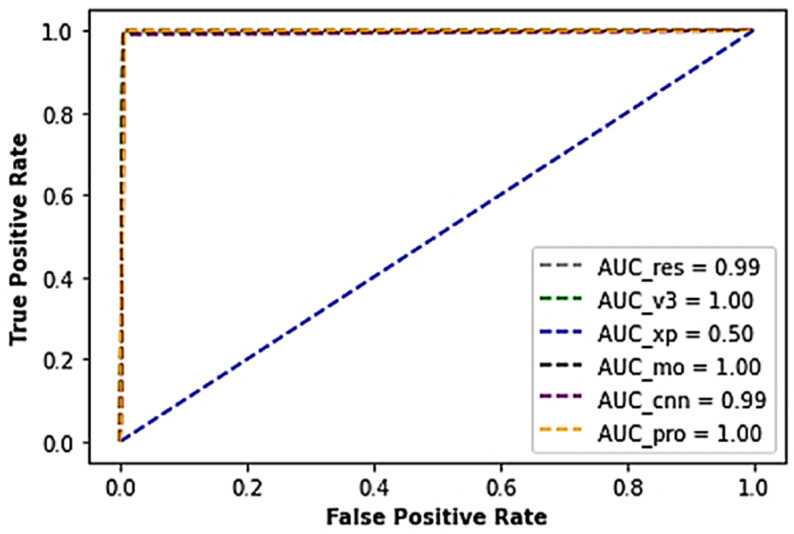
A cross-section of the AUC-ROC results of the industrial casting dataset.

In continuation, the voted model, the MobileNet, and InceptionV3 model produced an AUC score of about 100% each while the ResNet model outputted an AUC score of 99%, Xception 50% AUC score, and the typical CNN with a 99% AUC score (Figure 7).

**Figure 8:**
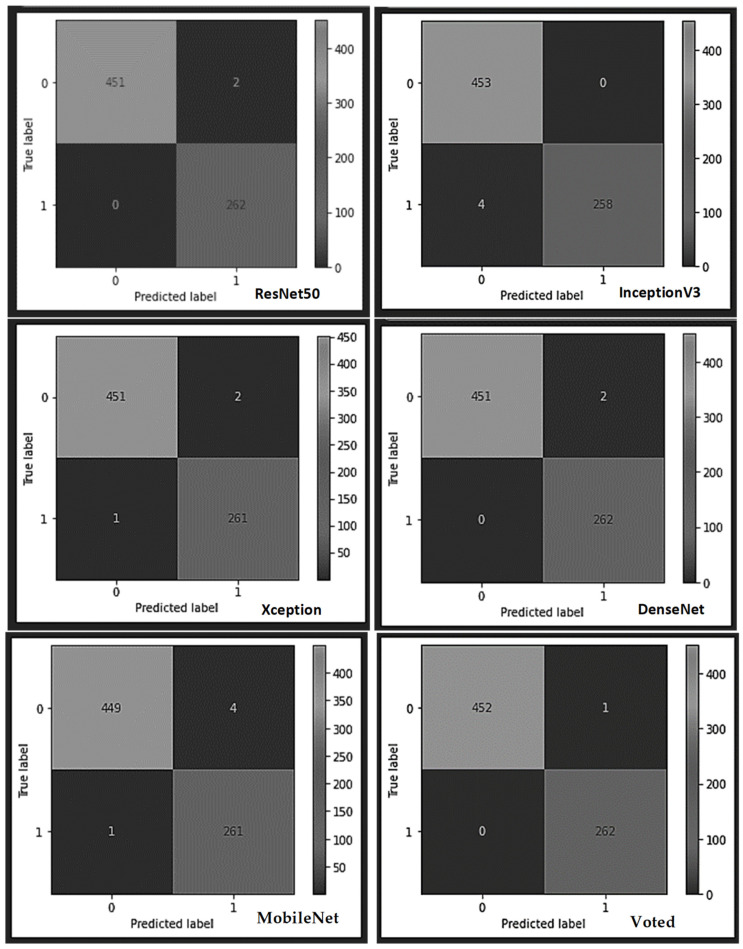
The various confusion matrices obtained from each learner using the impeller casting dataset.

**Figure 9:**
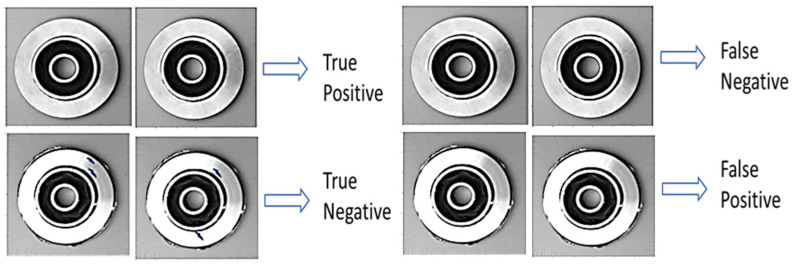
True positive, false negative, true negative, and false positive samples of the impeller casting products.

A breakdown of the outcome of each of the learner models and the voted model using the submersible pump impeller casting dataset (see the confusion matrix table in Figure 8 and Figure 9), shows that the built typical CNN model correctly classified all the 452 submersible pump impeller casting test samples as non-defect, except one sample misclassified as a defected sample. The model also identified all the defect test samples without any mistake, thereby yielding an overall superior performance against the other models. All the models yielded good results with negligible misrecognition except the InceptionV3, which misclassified four test samples belonging to the non-faulty class as faulty and the MobileNet, which misrecognized four samples as defected samples instead of non-defect samples.

Comparing our work with other similar studies shows that our proposed method outperformed the 98% accuracy score recorded by Kim, Ko, Choi and Kim [33], 98.79% score by Adibhatla, Chih, Hsu, Cheng, Abbod and Shieh [34], 98.1% by Bhattacharya and Cloutier [35], and 97.5 by Khalilian, Hallaj, Balouchestani, Karshenas and Mohammadi [36], in recognizing and differentiating defects and non-defects in a PCB dataset. Also, our method demonstrated a better performance in faulty and non-faulty piston recognition and classification compared to the 98.06% accuracy score recorded by [39,40] and an extended work performed by Tang, Zhu and Yuan [37] that produced a 99% accuracy score. On the submersible pump impeller casting dataset, our method outclassed the 95.5% accuracy score recorded by Lin, Yao, Ma and Wang [41], with a 99.9% accuracy, 9.96 × 10^−1^ sensitivity, 1.00 specificity, and 9.97 × 10^−1^ Cohen kappa scores, respectively.

Therefore, it can be concluded that our model in industrial implementation could achieve high accuracy with less latency. This could be an important feature for the implementation of such a system with real-time functionality. This work proposes an ensemble deep-leaning architectural framework based on a deep learning model architectural voting policy to compute and learn the hierarchical and high-level features in industrial artefacts.

## 6. Conclusions

A deep learning-driven industrial manufacturing product inspection method is presented in this paper. The study trained and ensembled different efficient models, including a crafted conventional CNN architecture with few layers and trainable parameters. An enhanced hard-voting policy was employed to select the best models to rapidly recognize and classify faulty and non-faulty manufactured products with respect to the set operating criteria.

The scope of this research deals with obtaining models that are more optimal, accurate and efficient in distinguishing defective products from non-defect ones in applications. In other words, the research focuses on producing a simple but highly efficient model that balances the tradeoff between model accuracy and latency. Therefore, the emphasis of the research is not on the optimality of the training process but on the optimality and suitable of the resulting models for deployment. However, we solved the model complexity and high cost of the model training by carefully crafting our base model, selecting the other competing model, training them separately, and using the voting policy to automatically determine the best models for each of the used datasets.

The operating principle of our proposed hard voting method is similar to the conventional hard voting method, which uses the probabilistic outcome of the various participating models to make predictions each time the models are run. However, our approach uses this technique to suggest the best model that can be deployed independently to make predictions without the contribution of the other models. In the experiments, the output weights of the voted models were used to determine the optimality and efficiency of the proposed method. An equal balance between the model optimality, accuracy, and efficiency was considered in the voting process using the various accuracy metrics and the weights produced from each competing model. For deployment and inferencing in applications, the voted models’ weights have to be low, although not the lowest; this ensures an equal balance between the production processing criteria. This critical factor makes the voted model more suitable for low-cost devices.

Three publicly available industrial datasets were adopted to train, test, and validate the proposed method, with remarkable results obtained that surpassed the existing compared methods. Our method produced models that are not only good in performance accuracy but optimal and efficient without sacrificing latency. One noticeable drawback observed in the work deals with the non-robustness of the PCB dataset. Thus, in future studies, more robust datasets will be included in the work to enhance the confidence level and performance of the proposed method.

## Figures and Tables

**Table 1:** The constructed base typical CNN model.

Layer Type	Output Shape	Parameters
Conv2D	None, 45, 45, 32	320
MaxPooling2D	None, 22, 22, 32	0
Conv2D	None, 11, 11, 64	18,496
MaxPooling2D	None, 5, 5, 64	0
Flatten	None, 1600	0
Dense	None, 128	204,928
Dense	None, 1	129

**Table 2:** Results achieved from the various learners with the industrial AC Piston dataset.

	Sensitivity	Specificity	Cohen_Kappa	Test Accuracy	Test Loss	Weights
CNN	8.50 × 10^−1^	9.60 × 10^−1^	8.22 × 10^−1^	9.29 × 10^−1^	2.66 × 10^−1^	5 MB
Inceptionv3	9.00 × 10^−1^	9.60 × 10^−1^	8.60 × 10^−1^	9.43 × 10^−1^	3.28 × 10^−1^	20 MB
Xception	0.00	1.00	0.00	7.14 × 10^−1^	5.04 × 10^−1^	18 MB
MobileNet	9.00 × 10^−1^	7.20 × 10^−1^	5.25 × 10^−1^	7.71 × 10^−1^	1.84	8 MB
ResNet50	1.00	0.00	0.00	2.86 × 10^−1^	3.76	21 MB
Den_Voted	9.00 × 10^−1^	1.00	9.28 × 10^−1^	9.71 × 10^−1^	5.24 × 10^−2^	12 MB

**Table 3:** Results achieved from the various learners with the industrial PCB dataset.

	Sensitivity	Specificity	Cohen_Kappa	Test Accuracy	Test Loss	Weights
CNN	2.89 × 10^−1^	1.78 × 10^−1^	−5.33 × 10^−1^	2.33 × 10^−1^	1.43	8 MB
Inceptionv3	9.89 × 10^−1^	9.56 × 10^−1^	9.44 × 10^−1^	9.72 × 10^−1^	1.34 × 10^−1^	22 MB
Xception	9.78 × 10^−1^	9.78 × 10^−1^	9.56 × 10^−1^	9.78 × 10^−1^	6.24 × 10^−2^	20 MB
MobileNet	1.00	8.89 × 10^−1^	8.89 × 10^−1^	9.44 × 10^−1^	1.45 × 10^−1^	11 MB
ResNet50	5.56 × 10^−2^	1.00	5.56 × 10^−2^	5.28 × 10^−1^	7.15	25 MB
Den_Voted	9.89 × 10^−1^	1.00	9.89 × 10^−1^	9.94 × 10^−1^	3.80 × 10^−2^	18 MB

**Table 4:** Results achieved from the various learners with the industrial casting dataset.

	Sensitivity	Specificity	Cohen_Kappa	Test Accuracy	Test Loss	Weights
Inceptionv3	1.00	9.96 × 10^−1^	9.94 × 10^−1^	9.97 × 10^−1^	7.55 × 10^−3^	28 MB
Xception	9.96 × 10^−1^	9.93 × 10^−1^	9.88 × 10^−1^	9.94 × 10^−1^	1.28 × 10^−2^	23 MB
Densenet	9.96 × 10^−1^	9.96 × 10^−1^	9.91 × 10^−1^	9.96 × 10^−1^	1.61 × 10^−2^	24 MB
MobileNet	9.96 × 10^−1^	9.96 × 10^−1^	9.91 × 10^−1^	9.96 × 10^−1^	1.33 × 10^−2^	13 MB
ResNet50	9.89 × 10^−1^	9.96 × 10^−1^	9.85 × 10^−1^	9.93 × 10^−1^	1.91 × 10^−2^	31 MB
CNN_voted	9.96 × 10^−1^	1.00	9.97 × 10^−1^	9.99 × 10^−1^	6.44 × 10^−3^	15 MB

## Data Availability

https://www.kaggle.com/datasets/satishpaladi11/mechanic-component-images-normal-defected, accessed on 12 August 2022. PCB defect dataset [53]. https://www.kaggle.com/datasets/ravirajsinh45/real-life-industrial-dataset-of-casting-product, accessed on 12 August 2022.
